# Usefulness of the Measurement of Serum Paraoxonase-1 Arylesterase Activity in the Diagnoses of COVID-19

**DOI:** 10.3390/biom12070879

**Published:** 2022-06-23

**Authors:** Xavier Gabaldó, Màrius Juanpere, Helena Castañé, Elisabet Rodríguez-Tomàs, Ana Felisa López-Azcona, Gerard Baiges-Gaya, Lourdes Castro, Enrique Valverde-Díaz, Aida Muñoz-Blázquez, Laura Giménez-Cuenca, Laura Felipo-Balada, Frederic Ballester, Isabel Pujol, Josep M. Simó, Antoni Castro, Simona Iftimie, Jordi Camps, Jorge Joven

**Affiliations:** 1 Laboratori de Referència Camp de Tarragona i Terres de l’Ebre, Hospital Universitari de Sant Joan, 43204 Reus, Spain; xgabaldo@lrsud.cat (X.G.); mjuanpere@lrsud.cat (M.J.); lcastro@lrsud.cat (L.C.); enrique.valverde@estudiants.urv.cat (E.V.-D.); aida.munoz@estudiants.urv.cat (A.M.-B.); fballester@lrsud.cat (F.B.); isabel.pujol@salutsantjoan.cat (I.P.); jmsimo@lrsud.cat (J.M.S.); 2 Unitat de Recerca Biomèdica, Institut d’Investigació Sanitària Pere Virgili, Hospital Universitari de Sant Joan, Universitat Rovira i Virgili, 43204 Reus, Spain; helena.castane@iispv.cat (H.C.); elisabet.rodriguez@urv.cat (E.R.-T.); gerard.baiges@iispv.cat (G.B.-G.); jorge.joven@salutsantjoan.cat (J.J.); 3 Department of Internal Medicine, Institut d’Investigació Sanitària Pere Virgili, Hospital Universitari de Sant Joan, Universitat Rovira i Virgili, 43204 Reus, Spain; anafelisa.lopez@salutsantjoan.cat (A.F.L.-A.); laura.gimenez@estudiants.urv.cat (L.G.-C.); laura.felipo@estudiants.urv.cat (L.F.-B.); antoni.castro@urv.cat (A.C.)

**Keywords:** biomarkers, COVID-19, paraoxonase-1, SARS-CoV-2

## Abstract

The development of inexpensive, fast, and reliable screening tests for COVID-19 is, as yet, an unmet need. The present study was aimed at evaluating the usefulness of serum arylesterase activity of paraoxonase-1 (PON1) measurement as a screening test in patients with different severity levels of COVID-19 infection. We included 615 COVID-19-positive patients who were classified as asymptomatic, mildly symptomatic, severely symptomatic, or fatally symptomatic. Results were compared with 50 healthy volunteers, 330 patients with cancer, and 343 with morbid obesity. Results showed PON1 activity greatly decreased in COVID-19 compared to healthy volunteers; a receiver operating characteristics plot showed a high diagnostic accuracy. The degree of COVID-19 severity did not influence PON1 levels. Our results indicated that PON1 determination was efficient for disease diagnosis, but not for prognosis. Furthermore, patients with obesity or cancer presented alterations similar to those of COVID-19 patients. As such, elevated levels of PON1 indicate the absence of COVID-19, but low levels may be present in various other chronic diseases. The assay is fast and inexpensive. We suggest that PON1 measurement could be used as an initial, high cut-off point screening method, while lower values should be confirmed with the more expensive nucleic acid amplification test.

## 1. Introduction

Coronavirus disease 2019 (COVID-19) continues to spread worldwide at dangerous levels, with high morbidity and mortality rates. Despite the relative success of vaccination campaigns in high-income countries, the majority of populations in large areas of the world do not have access to vaccines, or to effective measures to prevent virus transmission. Furthermore, variants such as omicron (PANGO identifier B.1.1.529) clearly demonstrated that new variants can appear that are more infective and more adept at escaping the action of existing vaccines. The current consensus in the scientific community is that COVID-19 will not be eradicated easily, and that populations would need to accommodate for the infection in the future. In this context, developing cheap, fast, and reliable screening tests remains a peremptory need.

Although antigen tests have demonstrated their efficacy in confirming infection in symptomatic patients, the gold standard method for the diagnosis of COVID-19 remains the detection of the viral messenger RNA by nucleic acid amplification tests (NAATs) [1]. However, these techniques are not without drawbacks. NAATs are expensive and require time and specialized laboratories that tend to be saturated or even overwhelmed during times of outbreak. Additionally, many countries cannot afford the cost of performing NAATs on several thousands of people. Conversely, antigen tests are unreliable in asymptomatic patients. We recently reported preliminary results suggesting that the measurement of paraoxonase-1 (PON1) arylesterase activity can be a good marker of COVID-19, with excellent sensitivity and specificity [2]. However, our study was performed in a limited number of hospitalized patients and, therefore, we cannot propose it as a blood test for application in the general population. In the present study, we aimed to investigate the usefulness of serum arylesterase activity of PON1 measurement in the evaluation of COVID-19 in a larger series of outpatients and hospitalized patients, with different levels of disease severity.

## 2. Materials and Methods

### 2.1. Study Design and Data Collection

This is a retrospective cohort study in sera from 615 patients with SARS-CoV2 infection, confirmed by NAAT or antigen test. They were recruited between April 2020 and September 2021 in Hospital Universitari de Sant Joan. Ours is a reference hospital with 352 beds located in Reus, a medium-sized city (100,000 inhabitants), located in Catalonia, on the northeast coast of Spain. Our hospital offers health coverage to the surrounding counties, comprising a population of about 250,000 mainly rural inhabitants, but including primary care centers and residences for the elderly. According to our care protocols, and in accordance with the recommendations of the health authorities [3], a NAAT or antigen test was performed on all patients who attended our hospital as outpatients seeking hospitalization or receiving attention in the Emergency Department. NAAT testing for COVID-19 took place, regardless of the reason for entry into the hospital or whether or not the patient had symptoms suggestive of COVID-19. The only exclusion criteria were if patients were younger than 18 years or had missing clinical data. Patients were classified according to the recommendations of the National Institute of Health [4], as follows: Asymptomatic infected patients were those who had a positive test with no symptoms of COVID-19; mildly symptomatic were those patients with a positive test and COVID-19-compatible symptoms who did not require hospitalization (oxygen saturation, SpO2, >94% respiratory rate <22 breaths/min and no dyspnea); severely symptomatic were those patients with a positive test and evidence of lower respiratory disease during clinical assessment (or imaging and who had an SpO2 <94% on room air at sea level and a respiratory rate >22 breaths/min); fatally symptomatic were those patients with a positive test who required high-flow oxygen therapy or mechanical ventilation (invasive or noninvasive) or extracorporeal membrane oxygenation. For statistical purposes, patients who died within 30 days post-diagnosis were considered within the fatally symptomatic group. We also recorded the days elapsed between obtaining a sample for PON1 determination and the beginning of the disease, as determined by the date-of-onset of symptoms or first-positive test. For comparison, we used sera from 50 healthy volunteers, 238 patients with breast cancer, 32 with lung cancer, 28 with head and neck cancer, 32 with rectal cancer, and 343 patients with morbid obesity (body mass index ≥ 40 kg/m^2^). Healthy volunteers were selected from an epidemiological study conducted in our area, and who had no clinical or biochemical evidence of renal insufficiency, liver disease, neoplasia, or neurological disorders. Patients with cancer and morbid obesity were recruited from clinical control subjects prior to receiving treatment with radiation therapy or bariatric surgery. Samples from all these subjects were collected before January 2020, and detailed descriptions of these populations have been reported previously [5,6,7,8,9].

Serum samples from all participants were stored in our Biobank at –80 °C until the time of analyses. This study was approved by the Comitè d’Ètica i Investigació en Medicaments (Institutional Review Board) of Institut d’Investigació Sanitària Pere Virgili (Resolutions CEIM 014/2017, 040/2018, amended on 16 April 2020, INFLAMET/15-04/4proj7 and OBESPAD/14·07-31proj3).

### 2.2. Laboratory Procedures

PON1 is an esterase and lactonase that hydrolyzes multiple substrates, and various assays have been described using some of these substrates. For the current study, we chose the determination of its arylesterase activity by measuring the hydrolysis of phenylacetate, because this substrate is not toxic, it is cheap and easy to obtain, and the assay is fast and is not affected by the enzyme’s genetic polymorphisms [10]. The activity was measured at 270 nm in a 9 mM Tris-HCl buffer, pH 8.0, and supplemented with 0.9 mM CaCl2, as previously reported [11]. The detection limit of the assay was 2.3 ± 1.1 U/L (mean ± SD, *n* = 3). The intra-assay coefficient of variation was 8% (*n* = 35). The inter-assay coefficient of variation was 9% (*n* = 12).

### 2.3. Statistical Analyses

All statistical calculations and graphic representations were performed with the Statistical Package for Social Sciences (SPSS 24.0, Chicago, IL, USA) and GraphPad Prism 6.01 (GraphPad Software, San Diego, CA, USA). Quantitative data are presented as medians and 95% confidence intervals and assessed for differences with the Mann–Whitney *U* test. The diagnostic accuracy of PON1 measurement was assessed by Receiver Operating Characteristics (ROC) curves and confusion matrices [12]. Sensitivity, specificity, positive predictive value (PPV), and negative predictive value (NPV) were calculated as previously reported [13].

## 3. Results

### 3.1. Characteristics of the COVID-19-Positive Patients in the Study

Raw data associated with this article are shown as Supplementary Materials. Asymptomatic patients were similar in age to those with mild symptomatic infection and younger than those with severe or fatal disease. Males composed the majority in all groups, except in the mild COVID-19 group. The most frequent symptoms were dyspnea, pneumonia, cough, and fever, and occurred most often in patients with severe and fatal disease. Vaccination rates on entry into hospital varied between 17% and 30% (Table 1).

**Figure 1:**
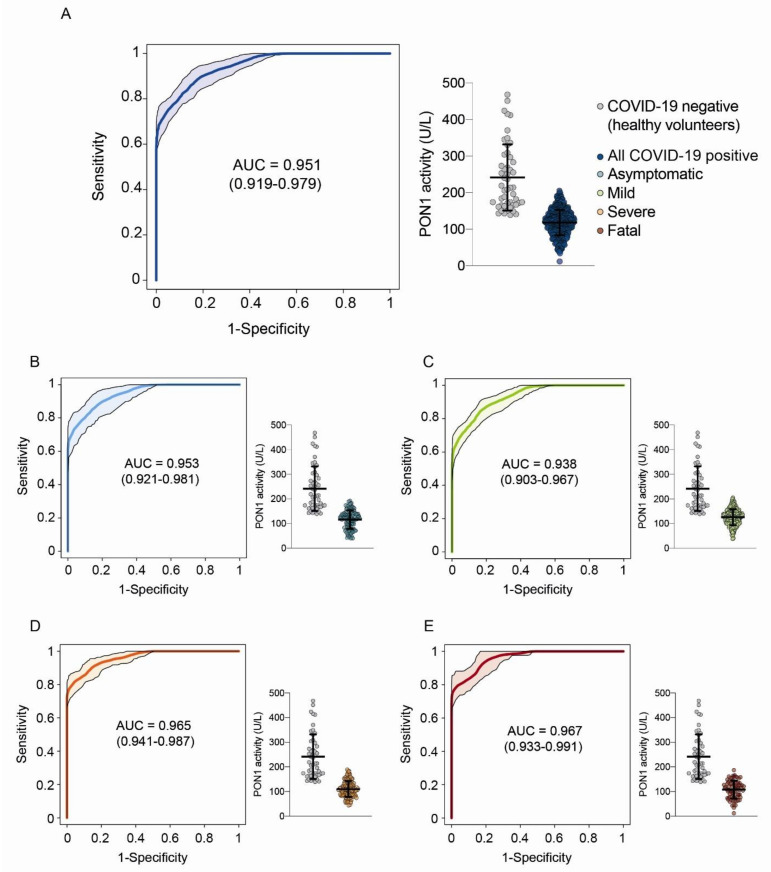
Serum paraoxonase-1 (PON1) activities and Receiver Operating Characteristics (ROC) curves comparing COVID-19-positive patients and the healthy controls; (**A**): All patients combined; (**B**): Asymptomatic patients; (**C**): Mild disease; (**D**): Severe disease; (**E**): Fatal disease. AUC: Area under the curve.

**Figure 2:**
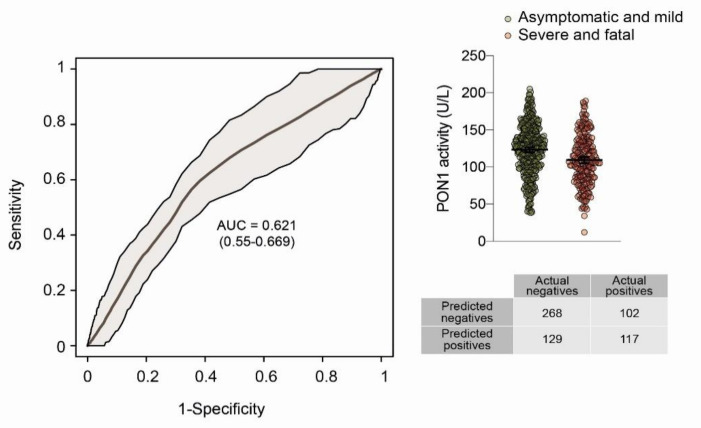
Serum paraoxonase-1 (PON1) and Receiver Operating Characteristics (ROC) curves comparing asymptomatic and mild *versus* severe and fatal COVID-19-positive patients. AUC: Area under the curve.

**Figure 3:**
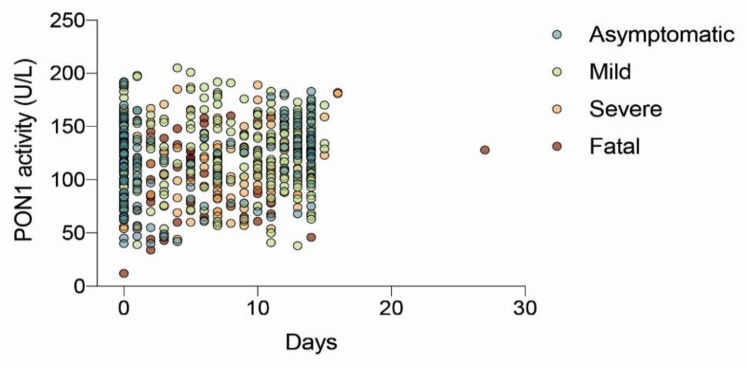
Serum paraoxonase-1 (PON1) activities in COVID-19 patients segregated according to the severity of symptoms and the days post-onset of the infection.

Serum PON1 arylesterase activity was 120.3 (59.5–176.1) U/L in COVID-19-positive patients and 213.3 (141.8–436.3) U/L in the healthy subjects (*p* < 0.001). The area under the curve (AUC) of the ROC plot was 0.951 (0.919–0.979) when comparing all COVID-19-positive patients with the healthy volunteers (Figure 1A). Similar results were obtained when COVID-19-positive patients were segregated according to the severity of their disease (Figure 1B–E). The best combination of sensitivity and specificity was obtained at PON1 = 161 U/L. We investigated the possibility of maximizing the sensitivity with the aim of using PON1 measurement as a screening test and found that at PON1 = 205 U/L, sensitivity was 100%, specificity was 56.0%, PPV was 96.6%, and NPV was 100.0% (Table 2). We also wished to distinguish the usefulness of PON1 measurement as a prognostic test but failed to observe differences in relation to the severity of the disease. Indeed, we found similar serum PON1 activities when comparing asymptomatic and mild versus severe and fatal disease; the AUC of the ROC curve was low, and the test misclassified a high percentage of patients (Figure 2). Finally, we wished to know whether there were any difference in serum PON1 values in relation to the days elapsing between the commencement of the infection and the measurement of PON1. No differences were observed regardless of the severity of the disease (Figure 3). We found no significant differences in PON1 activity between vaccinated and unvaccinated patients (115.3 (61.2–166.6; *n* = 146) U/L and 119.2 (58.2–178.2; *n* = 470) U/L, respectively). We also found no differences between patients who had comorbidities and those who had not: Cardiovascular disease (112.1 (83.0–136.7; *n* = 103) U/L and 113.3 (96.4–138.6; *n* = 57) U/L, respectively); diabetes (98.3 (76.2–142.3; *n* = 41) U/L and 114.7 (96.0–136.7; *n* = 119) U/L, respectively); liver disease (101.9 (71.3–151.7; *n* = 16) U/L and 113.9 (89.3–136.6; *n* = 144) U/L, respectively); obesity (non-morbid) (102.6 (82.7–133.2; *n* = 43) U/L and 115.1 (90.1–137.5; *n* = 117) U/L, respectively).

### 3.2. Paraoxonase-1 Activity Showed a Similar Trend toward a Decrease in COVID-19, Cancer, and Morbid Obesity Patients

**Figure 4:**
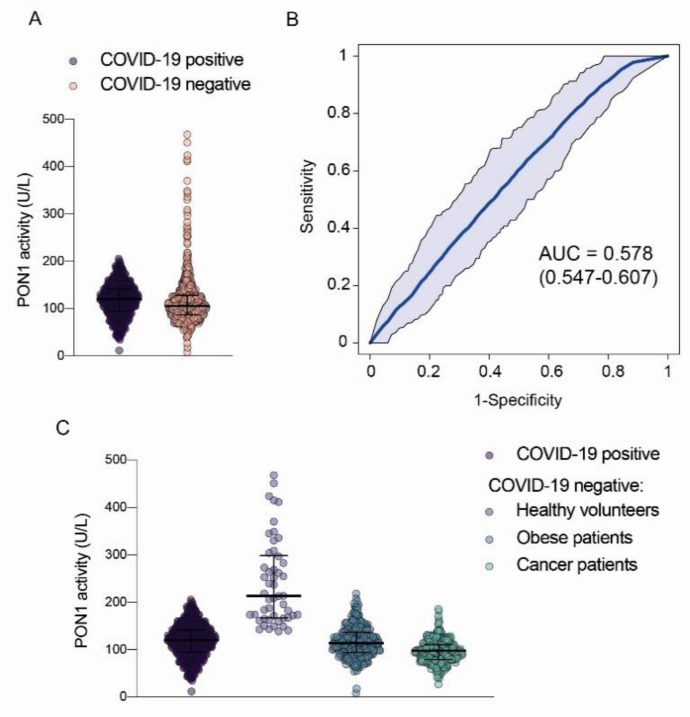
Serum paraoxonase-1 (PON1) activities in COVID-19-positive patients and all COVID-19-negative patients (obesity + cancer) and healthy volunteers combined (**A**); Receiver Operating Characteristics (ROC) curves comparing COVID-19-positive patients and COVID-19-negative patients (**B**); Serum paraoxonase-1 (PON1) activities in COVID-19-positive and COVID-19-negative patients separated according to their disease (**C**). AUC: Area under the curve.

PON1 activity was similar in COVID-19-positive patients and in patients with cancer or morbid obesity, when the latter group was considered together or when considered separately according to disease. The ROC curve showed that PON1 measurement was unable to segregate COVID-19-positive patients from subjects with the other diseases in the study group (Figure 4).

## 4. Discussion

PON1 is an enzyme synthesized mainly by the liver that is carried into the circulation bound to high-density lipoproteins. It can be internalized in peripheral cells and, thus, its protein expression is practically ubiquitous in almost all tissues. The enzyme is a lipoperoxide hydrolase that degrades lipoperoxides in lipoproteins and cells, and participates in the subject’s innate immune system and defense against oxidative stress [10,14]. Decreased serum PON1 activities have been reported in several diseases that have an infectious or inflammatory component, including bacterial infections [15,16,17], and non-communicable diseases such as chronic liver diseases, cardiovascular diseases, and cancer [10,14]. To elicit its physiological action, the active center of the enzyme needs to bind covalently to the substrate molecules; the outcome being that each enzyme molecule that binds to a lipoperoxide is inactivated and can no longer carry out its function. The result is a decreased enzyme activity [10]. An increase in lipid peroxidation therefore produces a decrease in enzymatic activity. Another cause that may explain the decrease in PON1 activity is that the composition of high-density lipoproteins is altered in COVID-19, with a decrease in cholesterol content and an increase in serum amyloid A [18]. These alterations are common in the acute phase of many diseases and can decrease PON1 activity by modifying its environment [15].

The present study, in a large series of patients, confirms our preliminary data indicating that serum PON1 activity is strongly decreased in COVID-19 infection [2] and demonstrates that this is an alteration that appears early in the course of the disease. Additionally, it is of note that the change is independent of whether or not the disease is severe. We might have expected a gradient of decreased enzyme activity depending on the severity of the disease, but our results suggest that if PON1 activity decreases as a consequence of oxidative stress and lipoprotein alterations secondary to infection, these changes are dissociated from the appearance of clinical symptoms. There are very few data on PON1 alterations in COVID-19 and on the role that this enzyme may play in the course of the disease. Proteomic studies have observed increased PON1 protein expression in high-density lipoproteins of COVID-19 patients [19,20,21] and suggested that this enzyme can be a marker of recovery [19]. Indeed, our preliminary study observed increased serum PON1 concentrations and decreased activities. This apparently contradictory result has also been observed in other infectious and non-communicable diseases [10,14]. The explanation is probably related to an attempt by the organism to increase synthesis of the enzyme to counteract the decrease in enzyme activity.

The results from the present study suggest that the widespread determination of serum PON1 arylesterase activity may be a useful parameter for the community-based diagnosis of COVID-19. The activity of this enzyme was greatly decreased in our patients, to levels approximately half of that of the healthy population, and regardless of whether or not the patient has concurrent COVID-19 symptoms, or even regardless of the severity of the symptoms. Perhaps this determination could be especially useful in low-income countries where millions of people have not yet been vaccinated, and the State cannot afford massive campaigns to detect positive patients using NAAT technology. Determination of PON1 arylesterase activity in serum is extremely simple and inexpensive. It only requires a spectrophotometer capable of reading at 270 nm and a pH-meter. If desired, the determination can be done in a plastic tube or on a plate, and it can be easily automated. The cost of reagents ought not to exceed a few cents. For example, the price of a 500 mL bottle of phenylacetate substrate is around EUR 40–50, and one plate of 96 determinations costs around EUR 10. The only other consumables needed are the reaction buffer and plastic tubes for the dilutions. The total cost must not be more than EUR 1 per sample. However, an important drawback is that observing low levels of this enzyme is not specific to COVID-19 infection, because similar levels of activity can be found in other chronic diseases such as morbid obesity or cancer. This limitation implies that the patient’s clinical history must be taken into account before interpreting the results. In addition, several medications, such as statins, fibrates, and aspirin may alter the arylesterase activity. Smoking is also an important modifying factor, although there is consensus that smokers are less likely to contract COVID-19, possibly due to an inhibitory effect of nicotine on the entry of the virus into the respiratory tract [22,23,24]. All of these limitations mean that there are many reasons other than COVID-19 to find a low serum PON1 activity. However, our results suggest that high activities are only found in people not infected by SARS-CoV-2. Indeed, we showed that, in our sample, the negative predictive value is 100% if a cut-off is set at 205 U/L. We suggest that the determination of PON1 be used as an initial screening method with a high cut-off point, with subsequent confirmation of the lower values using NAAT. This could save millions of euros.

One criticism that can be made of our study is that, currently, the epidemiological status of the COVID-19 infection has improved thanks to the predominance of the omicron strain where the symptoms are considerably milder, and the test we are proposing is no longer necessary. However, the omicron strain is much more transmissible than the previous ones and the number of infected individuals is considerably higher. Hence, the number of severe cases remain high, in absolute terms. In addition, although vaccines considerably restrict the numbers of severe cases, it is also true that vaccination rates are far from adequate in large areas of the world. As such, we believe that the determination of PON1 arylesterase activity can still be very useful. Other limitations of our study are that it was carried out in a single center, in a specific geographical area, with a specific lifestyle and in which the Caucasian ethnic group is the most abundant. Nevertheless, we believe that our data can stimulate further research, including more extensive national and international studies.

## Figures and Tables

**Table 1:** Demographic and clinical characteristics of studied patients.

Characteristic	Healthy Subjects (*n* = 50)	Asymptomatic COVID-19 (*n* = 113)	Mild COVID-19 (*n* = 284)	Severe COVID-19 (*n* = 143)	Fatal COVID-19 (*n* = 75)
Age (years)	76 (67–80)	50 (33–70)	45 (32–56)	73 (57–84)	72 (53–86)
Sex					
Men (%)	38 (76)	70 (61.9)	79 (27.7)	83 (58.0)	44 (61.1)
Women (%)	12 (24)	43 (38.1)	205 (72.2)	60 (42.0)	28 (38.9)
Symptoms					
Pneumonia (%)	-	-	12 (4.2)	37 (25.9)	17 (22.7)
Cough (%)	-	-	91 (32.0)	79 (55.2)	40 (53.3)
Fever (%)	-	-	115 (40.5)	85 (59.4)	39 (52.0)
Chills (%)	-	-	5 (1.8)	2 (1.4)	5 (6.7)
Dyspnea (%)	-	-	47 (16.5)	73 (51.0)	54 (72.0)
Vomiting (%)	-	-	15 (5.3)	9 (6.3)	5 (6.7)
Diarrhea (%)	-	-	36 (12.7)	20 (14.0)	11 (14.7)
Anosmia (%)	-	-	49 (17.2)	8 (5.6)	1 (1.3)
Ageusia (%)	-	-	40 (14.1)	10 (7.0)	2 (2.7)
Odynophagia (%)	-	-	24 (8.5)	7 (4.9)	2 (2.7)
Headache (%)	-	-	82 (28.9)	11 (7.7)	9 (12.0)
Anorexia/hyporexia (%)	-	-	8 (2.8)	8 (5.6)	3 (4.0)
Myalgia (%)	-	-	39 (13.7)	15 (10.5)	5 (6.7)
Arthralgia (%)	-	-	31 (10.9)	12 (8.4)	4 (5.3)
Respiratory failure (%)	-	-	5 (1.8)	6 (4.2)	12 (16.0)
Hospital stay (days)	-	-	-	9 (6–14)	13 (7–32)
Vaccine *					
Vaccinated (%)	-	32 (28.3)	50 (17.6)	43 (30.1)	20 (26.7)
1st dose (%)	-	11 (9.7)	11 (3.9)	16 (11.2)	9 (12)
2nd dose (%)	-	20 (17.7)	39 (13.7)	27 (18.9)	11 (14.7)
3rd dose (%)	-	1 (0.9)	0 (0)	0 (0)	0 (0)
Deceased (%)	-	-	-	-	31 (41.3)

* Samples from the healthy subjects were collected before the COVID-19 pandemic. Results are shown as medians and 95% confidence intervals (in parenthesis) or as number of cases and percentages.

**Table 2:** Diagnostic accuracy of paraoxonase-1 (PON1) measurement at two cut-off levels in the differentiation of patients with COVID-19 from non-COVID-19 patients.

PON1 Activity (U/L)		Diagnosis			Sensitivity (%)	Specificity (%)	PPV (%)	NPV (%)
		Negative	Positive	Total				
161	Negative	41	61	102	90.1 (87.4–92.3)	82.0 (68.1–91.0)	98.4 (96.9–99.2)	40.2 (30.8–50.4)
	Positive	9	554	564				
	Total	50	615	665				
205	Negative	28	0	28	100.0 (100.0–100.0)	56.0 (41.4–69.8)	96.6 (94.7–97.8)	100.0 (100.0–100.0)
	Positive	22	615	637				
	Total	50	615	665				

Results are shown at 161 U/L, which is the cut-off with the best combination of sensitivity and specificity, and at 205 U/L, which is the cut-off with 100% sensitivity. The numbers represent the number of cases or the percentages and 95% confidence intervals (in parenthesis). NPV: Negative predictive value. PPV: Positive predictive value. NAAT: Nucleic acid amplification test.

## Data Availability

The data presented in this study are available in the article and Supplementary Material.

## Supplementary Materials

The following supporting information can be downloaded at: https://www.mdpi.com/article/10.3390/biom12070879/s1. Excel file with the raw data of the study.
